# Is the brain a resource-cheapskate?

**DOI:** 10.3389/fnhum.2014.00857

**Published:** 2014-10-24

**Authors:** Liat Goldfarb, Avishai Henik

**Affiliations:** ^1^The Edmond J. Safra Brain Research Center for the Study of Learning Disabilities, University of HaifaHaifa, Israel; ^2^Department of Psychology, Zlotowski Center for Neuroscience, Ben-Gurion University of the NegevBeer Sheva, Israel

**Keywords:** selective attention, mental resources, attention under load, brain energy, limited resources

Any action requires energy. The body's muscles need energy in order to contract and the neurons in one's brain need energy in order to function. In the current paper we will discuss two presumably contradicting bodies of research. The first suggests that the brain invests minimal effort in high-demand tasks. The second suggests that we constantly spread our mental energy. We will refer to these findings and suggest some important rules that govern the consumption of mental energy.

## Mental energy is consumed economically

More than a century after the concept of mental energy or cognitive resource was introduced by James (1907), the discussion it creates is alive and well (however for criticism, see: Navon, 1984; Inzlicht et al., 2014). Resources can be treated as the power that allows us to perform cognitive tasks. Reduction in a resource allocation to a certain process can damage cognitive performance (Harvey, 2013). To characterize the notion of recourses some use the analogy of a fuel that feeds our mental processes (Gailliot et al., 2007). Others use the analogy of a computer with multiple processors and suggest that in order to perform cognitive tasks the brain allocates a finite number of mental processors. Accordingly, at a given time, resources are finite, dynamic, and divisible (Kurzban et al., 2013). Others suggest that resources can be both occupiable and depletable as analogy to a car and fuel which are respectively, occupiable and depletable travel resources (Harvey, 2013). One of the characteristics of resource allocation is that it is allocated economically.

It is clear that if one needs to proceed from point A to point B, the goal will be achieved quicker if one runs faster. However, in daily life you rarely see people completing their errands at a run from one place to another. People prefer to use their physical resources in an economical manner and they usually simply walk from place to place. This observation can be explained by the finding that an important feature of locomotion control is the minimization of metabolic cost per distance (e.g., Gutmann et al., 2006).

Since mental operations as well-consume resources, the brain should also treat its resources in an economical manner. Dror et al. (2005), for example, suggested that when the brain resources of the elderly decline, there is a qualitative change in the mental representation they use to perform a mental rotation task. Instead of spending a great proportion of energy on the same representation style they used when they were younger, they change their representation completely. According to Dror et al., older peoples' representation strategies are not as efficient as younger people's, but they use far fewer mental resources.

In two recent studies, we (Goldfarb and Henik, 2007, 2013) showed that even when it is clear that use of control can improve the ability to resolve conflicts in a task, participants are reluctant to use it. In fact, the operation of control depended on the level of expected conflict. When participants were able to utilize low resource strategies to perform a task, they did so, even if these strategies only led to good-enough performance rather than best performance. Another example of economic strategies is presented by Kool et al. (2010). They found that in a variety of decision making tasks, the participants decided to choose a course of action that was more economical and required less resource spending. For example, since switching between tasks is a difficult cognitive task, participants tend to choose a course of action with less task-switching demands.

Recently, several theories were raised in an attempt to explain why in high-demand cognitive tasks people tend to demonstrate economical behavior. For example, the intrinsic-cost perspective suggests that the brain has “*a set of preferences that favor a balance, over time, between cognitive exertion and cognitive disengagement or rest, an idea that originates in labor economics and which has been fruitfully applied to physical effort*” (Kool and Botvinick, 2013, p. 698). Similarly, according to the labor-leisure theory (e.g., Inzlicht et al., 2014), the brain tries to maintain a balance between cognitive labor and cognitive leisure or rest.

## An apparent contradiction: cheapskate or spender of labor energy?

Although the need for balance between labor and rest is obvious in regard to physical effort, it is important to remember that physical work and mental work do not necessarily operate in the same way. Think of a person who after a short walk decides to sit and rest on a bench. This man can now have a physical rest, his legs are no longer using the resources that were previously consumed by the walking task and no other organ will automatically compete for these available resources. For example, the person will not automatically begin waving his hands simply because there is extra energy in his system. In contrast, evidence suggests that the brain will indeed automatically start a mental action simply because there is energy available in the system. This evidence suggests that the brain is constantly at work, it spreads mental energy between tasks and frequently automatically uses the spare energy left over from one mental task to operate a new mental task. This evidence presumably describes cases that may not fit the economical labor perspective and in this section we will review them.

In recent years Nilli Lavie developed a theory of selective attention under load (e.g., Lavie, 1995, 2000, 2005). According to this theory, the processing of distractors can be decreased under a high perceptual load but not under a low perceptual load. High perceptual load consumes more resources, so there is less spare capacity left to perceive the distractor. However, according to this theory, processing of interfering distractors would be increased under a low perceptual load. Because the low perceptual load does not consume all the available resources, there is some free capacity left to be used by the distractors or by any other element in the field. It has been suggested that even involuntary or automatic processes might need resources in order to operate (e.g.: Paap and Ogden, 1981; Kahneman and Chajczyk, 1983; Tzelgov, 1997; Chajut and Algom, 2003). Hence the free capacity left form the main task can be used involuntarily in the process of perceiving the distractors. When the information provided by the distractor is incongruent with the information provided by the relevant stimulus, a conflict occurs, performance on the task is impaired, and RT is increased. Notice that this theory introduces a very spendthrift approach regarding resources consumed by demanding tasks, as it claims that “*any capacity not taken up in perception of task-relevant stimuli would involuntarily ‘spill over’ to the perception of task-irrelevant distractors*” (Lavie, 2005; p. 75). The notion of the spilling over of resources from one task to another demanding task is also compatible with an earlier claim made by Treisman (1969) regarding how the nervous system treats resources. “*It may be that the nervous system is forced to use whatever discriminative systems it has available, unless these are already fully occupied with other tests or inputs, so that we tend to use our perceptual capacity to the full on whatever sense data reach the receptors*.” (p. 296).

Another example of constant processing can be found in a paper by Mason et al. (2007). This paper suggested that our brain is always in action. When there are no external demands for action, the brain creates or possibly allows actions that are not based on external stimuli to intrude. Under these conditions the brain begins activating cortical regions associated with wandering thoughts and similar to Lavie's findings, as the external demand decreases, the activation of wandering thoughts increases. While wandering thoughts can be relaxing and might seem equivalent to a leisure phase, or even as a result of allowing the populations of excitatory neurons to spontaneously fire when the system changes to a rest phase, they might not always have a leasiring or relexing nature. In fact, Smallwood and Schooler (2006), in their review suggested that wandering thought can be laboring in nature, unpleasant and depressing. They also noted that, the moment the main task releases some resources these thoughts can automatically intrude on the system, and unfortunately they can be extremely resources consuming. “*Tasks that rely heavily on controlled processing will leave few working-memory resources available for mind wandering because off-task thinking also requires resources. Thus, mind wandering should be less likely to occur when the primary task is demanding and more likely to occur when the task is simple or automatic. Moreover, when mind wandering occurs in demanding tasks, it should be associated with deficits in performance because fewer resources are available to complete the primary task*” (p. 947).

## Combining the two notions: labor cheapskate and labor lover

We suggest that the cognitive system is both a labor cheapskate and a labor lover. Its functioning can revolve around the following rules: (a) The cognitive system is a resource saver with respect to a given labor process. In this way more resources are free and can thus be used in other processes. (b) The cognitive system is a resource spender as it uses some of the spare labor energy that it managed to save, to simultaneously operate other processes that are not required at the moment or do not seem absolutely necessary. These processes can be new labor processes. Note that those rules can theoretically be accommodated by the framework of recent theories on the intrinsic-cost perspective or the labor-leisure balance framework, if a constraint will be added in which the *sum* of all labor energy is being counted and then balanced with the rest-leisure criterion.

**Figure 1:**
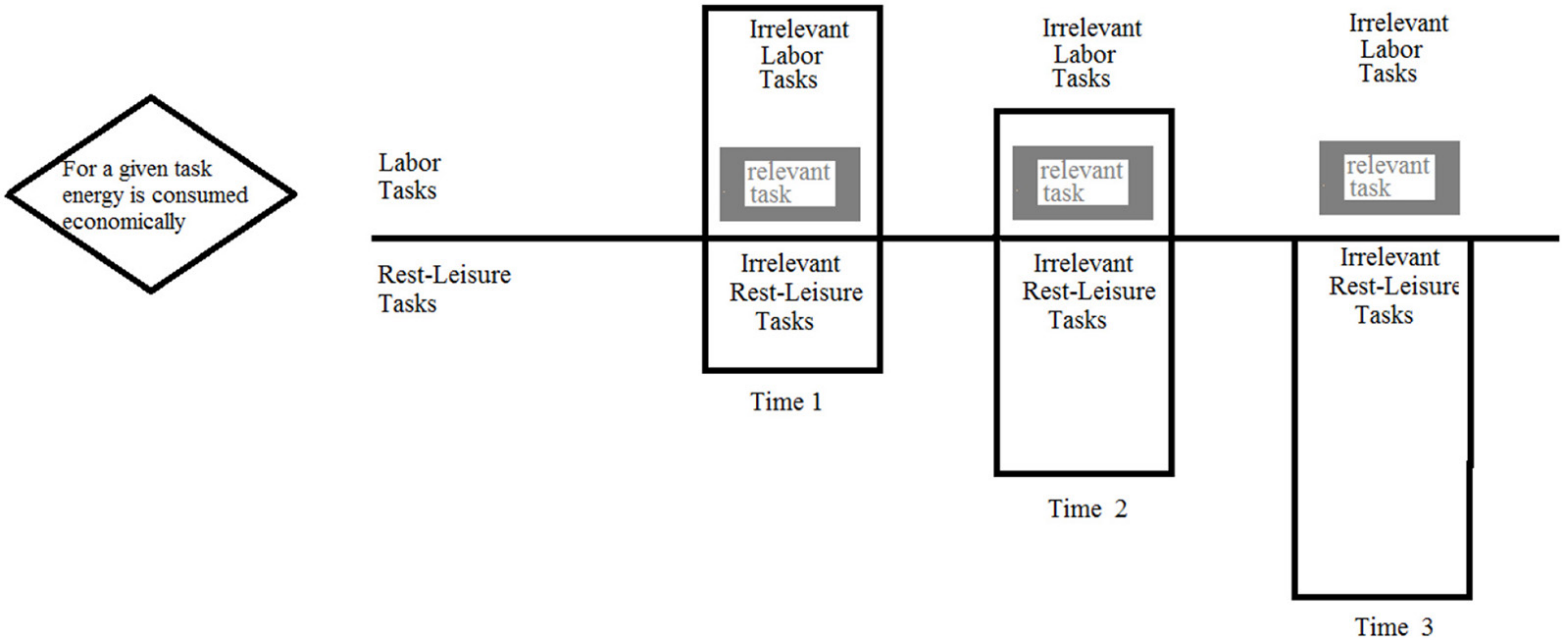
**An illustration of how the cognitive system may spread its energy**.

Figure 1 illustrates how these rules can work together. At a given Time 1, the cognitive system has a certain amount of energy to splurge on labor work. The system will consume the labor energy economically in order to perform a single task. The remaining labor energy can be automatically spread to other labor tasks. As we proceed along the time line a shift can occur in the total amount of labor energy (e.g., due to a shift in motivation, the need for leisure or rest). However, the total shift may not affect the consumption rules of the labor energy: for a given task energy is consumed economically so that the remaining labor energy can be spread to other labor tasks.

Why does the brain use labor energy economically with respect to a given task and thriftlessly in respect to presumably irrelevant tasks even when they are not fun or enjoyable? As noted, in the wandering thought theory, thoughts that are active in the absence of any external demand can be heavily engaging for the system and can contain unpleasant contents. Similarly, in the perception-under-load theory, the spending of spare resources will occur even when it actually damages performance. Since there is no control over the spillover of resources the outcome can be a behavioral impairment. However it is important to note that examples showing that free resources cause an unnecessary behavioral impairment are not a typical outcome of the squandering brain. Spillover of resources can have a positive outcome as it can lead to behavioral improvement (Cartwright-Finch and Lavie, 2006; Lavie, 2006). Similarly, engaging in thoughts driven by the wandering mind, even if they are not “restful thoughts,” can be useful for problem solving (see Smallwood and Schooler, 2006). In everyday life, using all the available resources simultaneously at a given moment is certainly useful. It is doubtful if humans would have survived if their perceptual resources had been focused only on a particular relevant object or on a restricted area of the perceptual field. It seems that it is best to focus on something and still be sensitive to other things in one's surroundings even if this involves hard work.

In conclusion, in the context of the labor energy available to the brain, the brain is a local cheapskate and simultaneously a global spender.

### Conflict of interest statement

The authors declare that the research was conducted in the absence of any commercial or financial relationships that could be construed as a potential conflict of interest.
